# Supplementary figures and images for: Mutations in microRNA-128-2-3p identified with amplification-free hybridization assay
Source: PLoS One. 2023 Aug 22;18(8):e0289556. doi: 10.1371/journal.pone.0289556 (PMC10443835; doi:10.1371/journal.pone.0289556)

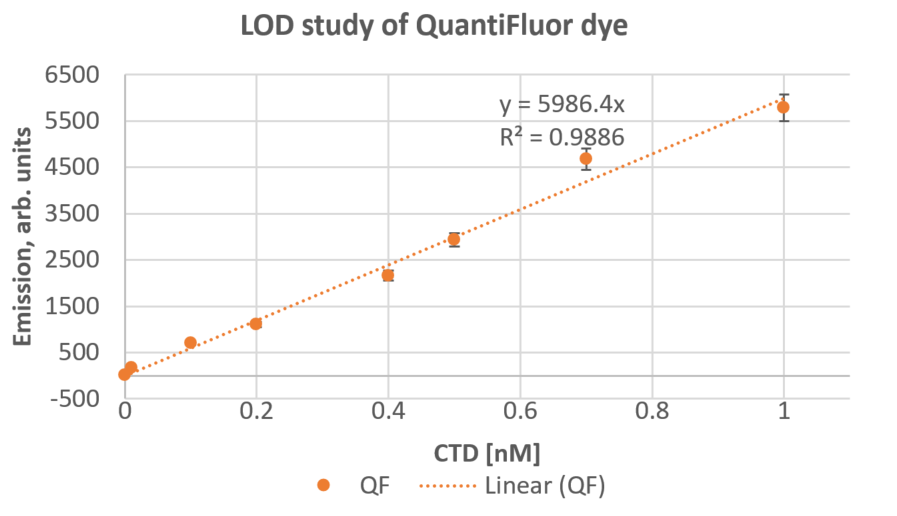

Supplement: S1 Fig — The LOD with signal to noise ratio > 3, was determined to be 2.2 pM for QuantiFluor, based on the calibration curves. (TIF) [file pone.0289556.s001.tif]
