# Supplementary material for: Mutations in microRNA-128-2-3p identified with amplification-free hybridization assay
Source: PLoS One. 2023 Aug 22;18(8):e0289556. doi: 10.1371/journal.pone.0289556 (PMC10443835; doi:10.1371/journal.pone.0289556)
Supplement: S1 Table — De-identified human colon cancer samples, along with 10 healthy controls (gender: 5 male and 5 female; Median age at sample (range) 44 (30–53)) were provided by Danish Biobank Regionernes Bio- og GenomBank (RBGB) Herlev, under National Videnskabsetisk Komité (National Research Ethics Committee) (NVK) number 21015520. (DOCX) [file pone.0289556.s002.docx]

Supporting Information S1 Table

**S1 Table. Clinical and demographical information of CRC patients.** De-identified human colon cancer samples, along with 10 healthy controls (gender: 5 male and 5 female; Median age at sample (range) 44 (30-53)) were provided by Danish Biobank Regionernes Bio- og GenomBank (RBGB) Herlev, under National Videnskabsetisk Komité (National Research Ethics Committee) (NVK) number 21015520.

| **Patient ID** | **Disease type** | **Age (years)** | **Gender** | **Stage** | **History of UC/CD*** | **UC/CD** | **Hemoglobin (Hgb) g/dL** | **ESR (mm/hour)** | **Race** | **Ethnicity** |
| --- | --- | --- | --- | --- | --- | --- | --- | --- | --- | --- |
| 15 | CRC | 30 | Male | 1 | 0 |  | 6,2 | 66 | Asian | Non Hispanic/ Non-Latino |
| 23 | CRC | 32 | Male | 1 | 0 |  | 7,4 | 25 | White | Non Hispanic/ Non-Latino |
| 41 | CRC | 54 | Male | 1 | 1 | UC | 8,8 | 11 | White | Non Hispanic/ Non-Latino |
| 24 | CRC | 61 | Female | 1 | 0 |  | 11 | 9 | not known | not known |
| 54 | CRC | 45 | Male | 1 | 1 | UC | 9,5 | 14 | White | Non Hispanic/ Non-Latino |
| 66 | CRC | 40 | Male | 2 | 1 | CD | 7,3 | 54 | White | Non Hispanic/ Non-Latino |
| 73 | CRC | 37 | Female | 1 | 0 |  | 8,2 | 13 | White | Non Hispanic/ Non-Latino |
| 111 | CRC | 62 | Female | 2 | 0 |  | 7 | 80 | White | Non Hispanic/ Non-Latino |
| 122 | CRC | 48 | Female | 1 | 0 |  | 11,3 | 66 | White | Non Hispanic/ Non-Latino |
| 132 | CRC | 52 | Male | 1 | 1 | UC | 12,5 | 43 | White | Non Hispanic/ Non-Latino |
| 140 | CRC | 35 | Male | remission | 0 |  | 15,4 | 4 | White | Non Hispanic/ Non-Latino |
| 118 | CRC | 31 | Male | 2 | 1 | UC | 11,3 | 66 | White | Non Hispanic/ Non-Latino |
| 156 | CRC | 44 | Male | 1 | 1 | UC | 10,6 | 50 | White | Non Hispanic/ Non-Latino |
| 189 | CRC | 52 | Male | remission | 0 |  | 12 | 32 | not known | not known |
| 221 | CRC | 38 | Female | remission | 0 |  | 14,6 | 11 | White | Non Hispanic/ Non-Latino |
| 212 | CRC | 50 | Male | remission | 0 |  | 17,8 | 2 | White | Non Hispanic/ Non-Latino |
| 234 | CRC | 34 | Female | 1 | 0 |  | 11 | 11 | Asian | Non Hispanic/ Non-Latino |
| 356 | CRC | 69 | Male | 1 | 0 |  | 8,4 | 23 | White | Non Hispanic/ Non-Latino |
| 401 | CRC | 60 | Male | 1 | 1 | UC | 7,2 | 60 | White | Non Hispanic/ Non-Latino |
| 408 | CRC | 54 | Male | 1 | 1 | CD | 9,9 | 49 | White | Non Hispanic/ Non-Latino |

Abbreviations: CRC: Colorectal cancer; *UC: Ulcerative colitis/CD: Crohn's disease; 0: No UC/DC, 1: Yes UC/DC
