# Supplementary material for: Mutations in microRNA-128-2-3p identified with amplification-free hybridization assay
Source: PLoS One. 2023 Aug 22;18(8):e0289556. doi: 10.1371/journal.pone.0289556 (PMC10443835; doi:10.1371/journal.pone.0289556)
Supplement: S2 Table — De-identified human colitis plasma samples, along with 10 healthy controls (gender: 5 male and 5 female; Median age at sample 44), were obtained from Stanford University (USA) under Stanford Institutional Review Board (IRB protocol 28427). (DOCX) [file pone.0289556.s003.docx]

Supporting Information S2 Table

**S2 Table. Clinical and demographical information of inflammatory bowel disease patients.** De-identified human colitis plasma samples, along with 10 healthy controls (gender: 5 male and 5 female; Median age at sample 44), were obtained from Stanford University (USA) under Stanford Institutional Review Board (IRB protocol 28427).

| **Patient ID** | **Disease type** | **Age (Years)** | **Gender** | **Diagnosis** | **Disease Activity** | **Hemoglobin (Hgb) g/dL** | **ESR (mm/hour)** | **Race** | **Ethnicity** |
| --- | --- | --- | --- | --- | --- | --- | --- | --- | --- |
| 124 | CD | 23 | M | Crohn's colitis | Flare | 7,8 | 6 | Not identified | Non Hispanic/  Non-Latino |
| 131 | CD | 30 | F | Crohn's ileocolitis | Flare | 12 | 2 | White | Non Hispanic/  Non-Latino |
| 137 | CD | 35 | F | Crohn's ileitis | Remission | 11,8 | 3 | African-American | Non Hispanic/  Non-Latino |
| 146 | CD | 51 | F | Crohn's colitis | Remission | 12,5 | 35 | White | Non Hispanic/  Non-Latino |
| 150 | CD | 25 | F | Crohn's ileocolitis | Remission | 11 | 17 | White | Non Hispanic/  Non-Latino |
| 158 | CD | 42 | M | Crohn's ileocolitis | Flare | 12,3 | 41 | White | Non Hispanic/  Non-Latino |
| 159 | CD | 31 | M | Crohn's ileocolitis | Flare | 10,6 | 58 | Hispanic | Non Hispanic/  Non-Latino |
| 164 | CD | 63 | M | Crohn's ileocolitis | Remission | 13,1 | 19 | White | Non Hispanic/  Non-Latino |
| 171 | CD | 41 | F | Crohn's colitis | Remission | 12,9 | 15 | African-American | Non Hispanic/  Non-Latino |
| 174 | CD | 31 | M | Crohn's colitis | Remission | 14,7 | 6 | White | Non Hispanic/  Non-Latino |
| 185 | CD | 57 | F | Crohn's ileitis | Flare | 9,6 | 72 | Not identified | Non Hispanic/  Non-Latino |
| 200 | CD | 68 | F | Crohn's colitis | Flare | 9,6 | 35 | White | Non Hispanic/  Non-Latino |
| 121 | UC | 68 | F | UC pancolitis | Flare | 9,6 | 62 | White | Non Hispanic/  Non-Latino |
| 122 | UC | 36 | F | UC pancolitis | Remission | 11,5 | 70 | White | Non Hispanic/  Non-Latino |
| 123 | UC | 60 | M | UC pancolitis | Flare | 10,5 | 65 | White | Non Hispanic/  Non-Latino |
| 133 | UC | 50 | M | UC pancolitis | Remission | 13,7 | 9 | Asian | Non Hispanic/  Non-Latino |
| 139 | UC | 37 | F | UC pancolitis | Remission | 13 | 9 | White | Non Hispanic/  Non-Latino |
| 140 | UC | 75 | F | UC pancolitis | Remission | 12,3 | 19 | White | Non Hispanic/  Non-Latino |
| 141 | UC | 23 | F | Ulcerative proctitis | Remission | 12,6 | 6 | White | Non Hispanic/  Non-Latino |
| 145 | UC | 37 | F | UC rectosigmoid | Flare | 12,3 | 19 | Not identified | Non Hispanic/  Non-Latino |
| 147 | UC | 26 | M | UC pancolitis | Remission | 14,4 | 6 | Not identified | Hispanic/Latino |
| 186 | UC | 28 | M | UC pancolitis | Flare | 8,3 | 39 | Patient declined | Non Hispanic/  Non-Latino |
| 191 | UC | 19 | F | UC pancolitis | Flare | 7,6 | 34 | White | Non Hispanic/  Non-Latino |
| 201 | UC | 60 | F | UC pancolitis | Flare | 12,8 | None | White | Non Hispanic/  Non-Latino |

Abbreviations: CD: Crohn's disease; UC: Ulcerative colitis; F: female; M: male.
