# Supplementary material for: Mutations in microRNA-128-2-3p identified with amplification-free hybridization assay
Source: PLoS One. 2023 Aug 22;18(8):e0289556. doi: 10.1371/journal.pone.0289556 (PMC10443835; doi:10.1371/journal.pone.0289556)
Supplement: S1 File — Mesoscopic model, temperature predictions, parameter optimizations, DNA:RNA optimization and experimental data. (DOCX) [file pone.0289556.s004.docx]

Supporting Information S1 File

**Mesoscopic modelling and parameter optimization of DNA:RNA hybrids**

### Model

The Peyrard-Bishop model describes the DNA helix through a Hamiltonian, which contains a Morse potential describing the hydrogen bonds that connect each base-pair and a harmonic potential describing the stacking interaction of adjacent base-pairs^1–4^ .

$U_{i,i+1}=\frac{k_{\alpha,\beta}}{2}\left( y_{i}-y_{i-1} \right)^{2}+D_{\alpha}\left( e^{-y/\lambda_{\alpha}}-1 \right)^{2},$ (1)

The equation above describes the interaction of the $i$th base pair with its nearest-neighbour, $i+1$. Where, $D_{\alpha}$ and $\lambda_{\alpha}$ describes the depth and width of the $i$th base-pair of type $\alpha$, respectively. An elastic constant $k_{\alpha,\beta}$, is used to describe the coupling between nearest neighbors base-pairs and the coordinate $y$ represents the relative displacements between the bases.

The sum for the Eq. (1) over all possible N base-pairs is carried out using its partition function:

$Z_{y}=\int_{y_{min}}^{y_{max}} dy_{1}\int_{y_{min}}^{y_{max}} dy_{2}\cdot\cdot\cdot\int_{y_{min}}^{y_{max}} dy_{N}\times\prod_{n=1}^{N} e^{-\beta U\left( y_{i},y_{i+1} \right)}$ (2)

where $\beta=1/{k_{B}}T$, $k_{B}$ is the Boltzmann constant and $T$ the absolute temperature. Subsequently, the integral over all possible configureurations of base pair displacements, $y_{i}$ is performed. From the partition function, Eq. (2) an adimensional index $\tau$ is derived and it is directly correlated with the experimental melting temperatures.

Furthermore, the average displacement, $\langle y_{m}\rangle$, at the $m$th position in the sequence can be derived from:

$\langle y_{m}\rangle=\frac{1}{Z_{y}}\int_{y_{min}}^{y_{max}} dy_{1}\int_{y_{min}}^{y_{max}} dy_{2}\cdot\cdot\cdot\int_{y_{min}}^{y_{max}} dy_{N}\times y_{m}\prod_{n=1}^{N} e^{-\beta U\left( y_{i},y_{i+1} \right)}$ (3)

### Temperature prediction

Considering a set $P=\{p_{1},p_{2},\cdot\cdot\cdot,p_{F}\}$ of Morse potentials, $D$, and stacking parameters, $k$, an index $\tau_{i}\left( P \right)$ is calculated for each sequence $i$ using the partition function, Eq. (2), for the Peyrard-Bishop Hamiltonian. Therefore, the melting temperature, $T_{i}^{'}\left( P \right)$ for each parameter set, $P$, is derived from the equation:

$T_{i}^{'}\left( P \right)=a_{0}\left( N \right)+a_{1}\left( N \right)\tau_{i}\left( P \right)$ (4)

where $N$ is the length of the sequence.

### Parameter optimization

The optimization process consists in minimizing the merit function

$\chi_{j}^{2}=\sum_{i=1}^{N} \left[ T_{i}^{'}\left( P_{j} \right)-T_{i} \right]^{2}$ (5)

where $T_{i}^{'}\left( P_{j} \right)$ is the calculated melting temperature from Eq. (4), $T_{i}$ is the experimental melting temperature and N is the number of melting temperatures in the dataset. The search for the minimum is made through a downhill simplex method^5^, and the procedure is made $j$ times. In each j, a new set of Morse potentials, $D$, and stacking parameters, $k$ is used. The problem of local minima is avoided repeating the minimization several times, starting over with different initial parameters following the procedures described in^2,6^. The final parameters shown here are those for the lowest overall $\chi^{2}$, and we estimate the parameter uncertainty from the standard deviations over the selected minimizations.

Additionally, we also use as a quality parameter the average prediction difference

$\langle\Delta T\rangle=\frac{1}{N}\sum_{i=1}^{N} \left| T_{i}^{'}-T_{i} \right|.$ (6)

### DNA:RNA optimization

The partition function was integrated up to 400 points in an interval between $y_{min}$= - 0.1 nm and $y_{max}$ = 20 nm with an eigenvalue cut-off of 10 (Eq. (22) of ^7^).

#### Seed parameters

In all optimizations the initial parameter $p_{i}$ is varied randomly in an interval

$p_{i}\in\left[ \left( 1-f \right)s_{i},\left( 1+f \right)s_{i} \right]$ (7)

that is, within a fraction $\pm f$ of a seed value $s_{i}$.

For instance, $f$=0.1, which results in the interval $\left[ 0.9s_{i},1.1s_{i} \right]$. As initial parameters for Morse potentials, $D$, and stacking parameters, $k$, were used generic parameters^6^. The minimized Morse potentials and stacking parameters are displayed in Table A.

**Table A.** **DNA:RNA stacking (k) and Morse (D) parameters at 100 mM [Na^+^]**.

| **k (eV/nm^2^)** | | **D (meV)** |
| --- | --- | --- |
| dArU-dArU 0.75(0.59) | dArU-dCrG 7.09(1.94) | dArU 20.75(12.42) |
| dArU-dGrC 2.69(0.75) | dArU-dTrA 4.42(1.63) | dCrG 64.23(12.46) |
| dCrG-dArU 3.77(1.30) | dCrG-dCrG 2.48(0.63) | dGrC 68.87(14.07) |
| dCrG-dGrC 1.51(0.51) | dCrG-dTrA 2.67(0.66) | dTrA 40.69(12.51) |
| dGrC-dArU 2.24(0.56) | dGrC-dCrG 2.07(0.69) |  |
| dGrC-dGrC 1.47(0.73) | dGrC-dTrA 1.93(0.72) |  |
| dTrA-dArU 1.34(0.90) | dTrA-dCrG 2.98(0.55) |  |
| dTrA-dGrC 2.34(0.88) | dTrA-dTrA 2.47(0.76) |  |

Estimated uncertainty is shown in compact notation.

#### Experimental data

The dataset of melting temperatures has 73 DNA:RNA hybrids sequences between 6-and 21-mer length at concentration of 100 mM [Na^+^] and 8 $\mu$M of strand. Table B shows all the sequences with the experimental and predicted melting temperatures.

**Table B.** **Sequences of DNA:RNA hybrids with their measured (**$\boldsymbol{T}_{\boldsymbol{exp}}$**) and predicted melting temperatures (**$\boldsymbol{T}_{\boldsymbol{pre}}$**).**

| $\boldsymbol{5'\to3'}$ | $\boldsymbol{3'\to5'}$ | $\boldsymbol{T}_{\boldsymbol{exp}}$ | $\boldsymbol{T}_{\boldsymbol{pre}}$ |
| --- | --- | --- | --- |
| r(GGUCGC) | d(CCAGCG)*^e^* | 27.2 | 26.52 |
| r(CGGACC) | d(GCCTGG)*^e^* | 26.1 | 25.41 |
| r(GCCGUGAG) | d(CGGCACTC)*^e^* | 41.2 | 40.92 |
| r(GAGCCGUG) | d(CTCGGCAC)*^e^* | 41.5 | 40.92 |
| r(GUCAGACU) | d(CAGTCTGA)*^e^* | 29.7 | 29.17 |
| r(GACAGUCU) | d(CTGTCAGA)*^e^* | 30.1 | 29.17 |
| r(GAACUGCC) | d(CTTGACGG)*^e^* | 33.5 | 31.96 |
| r(GGCAGUUC) | d(CCGTCAAG)*^e^* | 33.8 | 31.86 |
| r(GCACAGCC) | d(CGTGTCGG)*^a^* | 35.6 | 39.90 |
| d(GCACAGCC) | r(CGUGUCGG)*^a^* | 37.2 | 40.69 |
| r(CCUUCCCUU) | d(GGAAGGGAA)*^a^* | 20.5 | 23.42 |
| r(UUCCCUUCC) | d(AAGGGAAGG)*^a^* | 14.9 | 22.74 |
| d(CCTTCCCTT) | r(GGAAGGGAA)*^a^* | 44.8 | 41.57 |
| d(TTCCCTTCC) | r(AAGGGAAGG)*^a^* | 44.2 | 42.03 |
| r(GCCAGUUAA) | d(CGGTCAATT)*^d^* | 30.6 | 29.92 |
| r(GCGAUCGGA) | d(CGCTAGCCT)*^e^* | 43.5 | 41.82 |
| r(GCCAGUAGG) | d(CGGTCATCC)*^e^* | 42.6 | 42.87 |
| r(GCUCUCUGGC) | d(CGAGAGACCG)*^a^* | 40.8 | 42.33 |
| d(GCTCTCTGGC) | r(CGAGAGACCG)*^a^* | 50.9 | 51.22 |
| r(GAAGAGAAGC) | d(CTTCTCTTCG)*^b^* | 46.9 | 43.10 |
| d(GAAGAGAAGC) | r(CUUCUCUUCG)*^b^* | 23.7 | 22.87 |
| r(GUUCAAUACG) | d(CAAGTTATGC)*^e^* | 27.5 | 28.24 |
| r(AGGAUGACCG) | d(TCCTACTGGC)*^e^* | 45.9 | 45.35 |
| r(CGCUUGUUAC) | d(GCGAACAATG)*^e^* | 33.1 | 29.68 |
| r(GUAACAAGCG) | d(GTGAACAATG)*^e^* | 39.2 | 39.10 |
| r(CACUUGUUAC) | d(GTGAACAATG)*^e^* | 28.1 | 25.17 |
| r(AAUCUGGCCA) | d(TTAGACCGGT)*^e^* | 42.8 | 40.06 |
| r(AUGGCUCCAA) | d(TACCGAGGTT)*^e^* | 40.1 | 40.06 |
| r(GGGGAACAAGG) | d(CCCCTTGTTCC)*^e^* | 54.3 | 52.93 |
| r(UUCACCUGGUC) | d(AAGTGGACCAG)*^e^* | 45.3 | 40.92 |
| d(TCCCTCCTCTCC) | r(AGGGAGGAGAGG)*^a^* | 61.4 | 60.89 |
| r(GGCAGGAAUCCG) | d(CCGTCCTTAGGC)*^e^* | 56.8 | 54.94 |
| r(GGAAUCAGGCCG) | d(CCTTAGTCCGGC)*^e^* | 56.3 | 54.94 |
| r(UAUCUUCCGAAU) | d(ATAGAAGGCTTA)*^e^* | 30.2 | 32.46 |
| r(UAUCCUUCGAAU) | d(ATAGGAAGCTTA)*^e^* | 29.6 | 32.47 |
| r(AAUGGAUUACAA) | d(TTACCTAATGTT)*^e^* | 36.3 | 34.51 |
| r(AUUGGAUACAAA) | d(TAACCTATGTTT)*^e^* | 36.2 | 34.50 |
| r(CCUGGAAUCCAA) | d(GGACCTTAGGTT)*^e^* | 48.2 | 46.79 |
| r(GGCUCAAUUGAC) | d(CCGAGTTAACTG)*^e^* | 45.2 | 43.38 |
| r(CGGCCUUGAUCC) | d(GCCGGAACTAGG)*^e^* | 51.9 | 49.57 |
| r(CGGAUUCCUGCC) | d(GCCTAAGGACGG)*^e^* | 50.3 | 49.58 |
| r(UCCGAAUUAUCU) | d(AGGCTTAATAGA)*^e^* | 35.8 | 32.47 |
| r(AGAUAAUUCGGA) | d(TCTATTAAGCCT)*^e^* | 35.5 | 37.62 |
| r(GCUUCUCUCUUC) | d(CGAAGAGAGAAG)*^e^* | 31.5 | 31.17 |
| r(GAAGAGAGAAGC) | d(CTTCTCTCTTCG)*^e^* | 54.0 | 51.39 |
| r(UCGUUCUUGUCU) | d(AGCAAGAACAGA)*^e^* | 36.4 | 35.71 |
| r(AGACAAGAACGA) | d(TCTGTTCTTGCT)*^e^* | 47.6 | 47.45 |
| r(AUUGGAUACAAA) | d(TAACCTATGTTT)*^d^* | 35.5 | 34.50 |
| d(GAGCTCCCAGGC) | r(CUCGAGGGUCCG)*^a^* | 60.3 | 57.00 |
| r(GAGCUCCCAGGC) | d(CTCGAGGGTCCG)*^a^* | 56.7 | 57.95 |
| r(UCCCUCCUCUCC) | d(AGGGAGGAGAGG)*^a^* | 43.4 | 45.41 |
| r(GUUAGCGUUACGC) | d(CAATCGCAATGCG)*^e^* | 45.0 | 46.20 |
| r(GCGUUUACGUAGC) | d(CGCAAATGCATCG)*^e^* | 47.8 | 46.19 |
| r(UCACGUAGUCGUAU) | d(AGTGCATCAGCATA)*^e^* | 49.8 | 49.57 |
| r(UGUACGUCACAACUA) | d(ACATGCAGTGTTGAT)*^a^* | 49.2 | 49.24 |
| r(UAUACAAGUUAUCUA) | d(ATATGTTCAATAGAT)*^a^* | 35.9 | 34.05 |
| r(CGACUAUGCAAAAAC) | d(GCTGATACGTTTTTG)*^a^* | 47.3 | 48.47 |
| d(TAGTTATCTCTATCT) | r(AUCAAUAGAGAUAGA)*^a^* | 45.4 | 43.28 |
| d(TGTACGTCACAACTA) | r(ACAUGCAGUGUUGAU)*^a^* | 50.6 | 50.64 |
| d(TATACAAGTTATCTA) | r(AUAUGUUCAAUAGAU)*^a^* | 35.2 | 35.29 |
| d(CGACTATGCAAAAAC) | r(GCUGAUACGUUUUUG)*^a^* | 39.0 | 41.13 |
| r(UAGUUAUCUCUAUCU) | d(ATCAATAGAGATAGA)*^a^* | 34.9 | 34.45 |
| d(CGACTATGCAAGTAC) | r(GCUGAUACGUUCAUG)*^c^* | 45.1 | 48.84 |
| d(CGCAAAAAAAAAACGC) | r(GCGUUUUUUUUUUGCG)*^a^* | 28.7 | 30.70 |
| r(CGCAAAAAAAAAACGC) | d(GCGTTTTTTTTTTGCG)*^a^* | 50.2 | 51.44 |
| d(GGACCGGAAGGTACGAG) | r(CCUGGCCUUCCAUGCUC)*^c^* | 57.0 | 57.99 |
| d(CTCGTACCTTCCGGTCC) | r(GAGCAUGGAAGGCCAGG)*^a^* | 64.8 | 65.29 |
| r(CUCGUACCUUCCGGUCC) | d(GAGCATGGAAGGCCAGG)*^a^* | 56.0 | 57.99 |
| r(CUCGUACCUUUCCGGUCC) | d(GAGCATGGAAAGGCCAGG)*^a^* | 56.8 | 55.89 |
| d(CTCGTACCTTTCCGGTCC) | r(GAGCAUGGAAAGGCCAGG)*^a^* | 65.2 | 64.61 |
| d(CTCGTACCATTCCGGTCC) | r(GAGCAUGGUAAGGCCAGG)*^c^* | 63.7 | 64.22 |
| r(GCCGAGGUCCAUGUCGUACGC) | d(CGGCTCCAGGTACAGCATGCG)*^a^* | 68.1 | 66.04 |
| d(GCCGAGGTCCATGTCGTACGC) | r(CGGCUCCAGGUACAGCAUGCG)*^a^* | 68.2 | 64.98 |

Melting temperatures were predicted by the developed Peyrard-Bishop model. Main strands are shown on top of each row in 5'$\to$3' direction, while complementary strands are in the bottom of the same row in 3'$\to$5' direction. The d and r before the oligonucleotide mean DNA and RNA sequence, respectively. The final quality parameters of the optimization were $\langle\Delta T\rangle$ = 1.65 °C and $\chi^{2}$ = 330.58 °C^2^. *^a^* Sequences collected from^8^. *^b^* Sequences collected from^9^. *^c^* Sequences collected from^10^. *^d^* Sequences collected from^7^. *^e^* Sequences collected from^11^.

### References

1 G. Weber, N. Haslam, J. W. Essex and C. Neylon, *J. Phys. Condens. Matter*, 2009, **21**, 034106.

2 E. de Oliveira Martins, V. Basílio Barbosa, G. Weber, E. de Oliveira Martins, V. Basílio Barbosa and G. Weber, *Chem. Phys. Lett.*, 2019, **715**, 14–19.

3 I. Domljanovic, M. Taskova, P. Miranda, G. Weber and K. Astakhova, *Commun. Chem.*, , DOI:10.1038/s42004-020-00362-5.

4 N. Sugimoto, S. ichi Nakano, M. Katoh, A. Matsumura, H. Nakamuta, T. Ohmichi, M. Yoneyama and M. Sasaki, *Biochemistry*, 1995, **34**, 11211–11216.

5 W. H. Press, B. P. Flannery, S. A. Teukolsky and . T. Vetterling, *Numerical recipes in C—the art of scientific computing*, Cambridge University Press, 1988, vol. 73.

6 G. Weber, *Nucleic Acids Res.*, 2013, **41**, e30.

7 S. I. Nakano, M. Fujimoto, H. Hara and N. Sugimoto, *Nucleic Acids Res.*, 1999, **27**, 2957–2965.

8 E. A. Lesnik and S. M. Freier, *Biochemistry*, 1995, **34**, 10807–10815.

9 J. I. Gyi, G. L. Conn, A. N. Lane and T. Brown, *Biochemistry*, 1996, **35**, 12538–12548.

10 A. M. Kawasaki, M. D. Casper, S. M. Freier, E. A. Lesnik, M. C. Zounes, L. L. Cummins, C. Gonzalez and P. Dan Cook, *J. Med. Chem.*, 1993, **36**, 831–841.

11 D. Banerjee, H. Tateishi-Karimata, T. Ohyama, S. Ghosh, T. Endoh, S. Takahashi and N. Sugimoto, *Nucleic Acids Res.*, 2020, **48**, 12042–12054.
