# Supplementary material for: Mutations in microRNA-128-2-3p identified with amplification-free hybridization assay
Source: PLoS One. 2023 Aug 22;18(8):e0289556. doi: 10.1371/journal.pone.0289556 (PMC10443835; doi:10.1371/journal.pone.0289556)
Supplement: S2 File — Sequences of purchased oligonucleotides, synthesized oligonucleotide, characterization and purification. (DOCX) [file pone.0289556.s005.docx]

Supporting Information S2 File

## Oligonucleotides

### Purchased oligonucleotides

Biotinylated DNA and miR-128-2-3p were all purchased from IDT. Sequences are shown in Table A. miR128-2-3p target and capturing probes (C1-C14) were used for miRNA SNP bead-assay detection. Model miR128 targets (M1-M7) and capturing probes (C1-C7) were used for UV melting studies (Tm). The CTD binding linker was synthesized by solid-phase oligonucleotide synthesis.

**Table A. Sequences of purchased oligonucleotides.**

| Name | $\mathbf{5}\boldsymbol{'\to}\mathbf{3}\mathbf{'}$ | Name | $\mathbf{5}\boldsymbol{'\to}\mathbf{3}\mathbf{'}$ |
| --- | --- | --- | --- |
| miR-128-2-3p | r(ucacagugaaccggucucuuu) | C11 | Bt-Sp9-d(AAA+T+AGACC+GGT) |
| C1 (wt) | Bt-Sp9-d(AAAGAG+AC+CG+GT) | C12 | Bt-Sp9-d(AAACAGA+CCG+GT) |
| C2 | Bt-Sp9-d(TAAGAG+AC+CG+GT) | C13 | Bt-Sp9-d(AAAAAG+A+CCG+GT) |
| C3 | Bt-Sp9-d(CAAGAG+AC+CG+GT) | C14 (scr) | Bt-Sp9-d(AGCGCGGATAAA) |
| C4 | Bt-Sp9-d(GAAGAG+AC+CG+GT) | miR-128 M1 | r(ACCGGUCUCUUU) |
| C5 | Bt-Sp9-d(ATAGA+GACC+G+GT) | miR-128 M2 | r(ACCGGUCUCUUA) |
| C6 | Bt-Sp9-d(ACAGA+GACC+G+GT) | miR-128 M3 | r(ACCGGUCUCUUG) |
| C7 | Bt-Sp9-d((+AGA+GAGA+CCGGT) | miR-128 M4 | r(ACCGGUCUCUUC) |
| C8 | Bt-Sp9-d(AAT+GAG+A+CCGGT) | miR-128 M5 | r(ACCGGUCUCUAU) |
| C9 | Bt-Sp9-d(AAC+GAGA+CCGGT) | miR-128 M6 | r(ACCGGUCUCUGU) |
| C10 | Bt-Sp9-d(AAG+GAGA+CCGGT) | miR-128 M7 | r(ACCGGUCUCUCU) |

Bt= Biotin; Sp9 = Spacer C_9_H_18_; scr = scramble control; d = deoxyribonucleotides; r = ribonucleotides.

### Oligonucleotide synthesis

The LNA/DNA linker was synthesized in 1 μmol scale using solid support (CPG 1000 Å, Sigma Aldrich) on an Expedite^TM^ Nucleic Acid Synthesis System with reagents purchased from Sigma Aldrich: TCA Deblock, DCI activator 0.25 M, Oxidizer 0.02 M, Cap A and Cap B. Commercial phosphoramidites from Sigma Aldrich (dA(Bz), dC(Bz), dG(ib), dT) were all prepared in 0.07M solutions using dry acetonitrile (ACN). LNA phosphoramidites (indicated with ‘+’ in table below) from Qiagen (LNA-A(bz), LNA-mC(Bz), LNA-G(dmf)) were added using a hand coupling method: 25 mg LNA phosphoramidite was dissolved with 400 μl dry ACN (+C: 200 μl DCM+200 μl ACN) and 600 μl DCI Activator in a 1 ml syringe, mixed and added to the column with 50μl/min. The oligonucleotide was cleaved from solid support using 28-30% ammonia solution from Sigma Aldrich at 55 °C for 12h. The identity of the oligonucleotide was established by mass spectrometry (MS) using an Autoflex speed MALDI-TOF mass spectrometer (Bruker Daltonics, Hamburg, Germany). Samples were co-spotted with 3-Hydroxypicolinic acid as matrix on a MTP AnchorChip target plate for the analysis. The obtained mass spectra were recorded by flexControl 3.4 (Bruker Daltonics, Germany) software. The oligonucleotide was purified on an Ultimate 3000 UHPLC (Dionex, Sunnyvale, CA, USA) using a DNA-Pac RP (Thermo Fisher Scientific, Waltham, MA, USA) column (4 µm, 3.0 × 100 mm2) with a gradient of 5–25% buffer B in A over 30 min at 60 ℃. Buffer A: 0.05 M TEAA; Buffer B: 25% buffer A in acetonitrile. Peaks were monitored at 260 nm. The final pure oligonucleotide (87.6% pure) had a yield of 8% (80.6 nmol), MS calc. 9487.2 g/mol, MS obs. 9487.106 m/z. HPLC and MALDI spectra are shown below.

- 1. **Characterization: MALDI and HPLC**

| **Name** | **Sequence** $\boldsymbol{5}^{\boldsymbol{'}}\boldsymbol{\to3'}$ | **MS calc. [g/mol]** | **MS obs. [m/z]** |
| --- | --- | --- | --- |
| Linker | T+C+A+CT+GT+GATTTTGAT+GGG+AATAC+CAGACC | 9487.2 | 9487.106 |


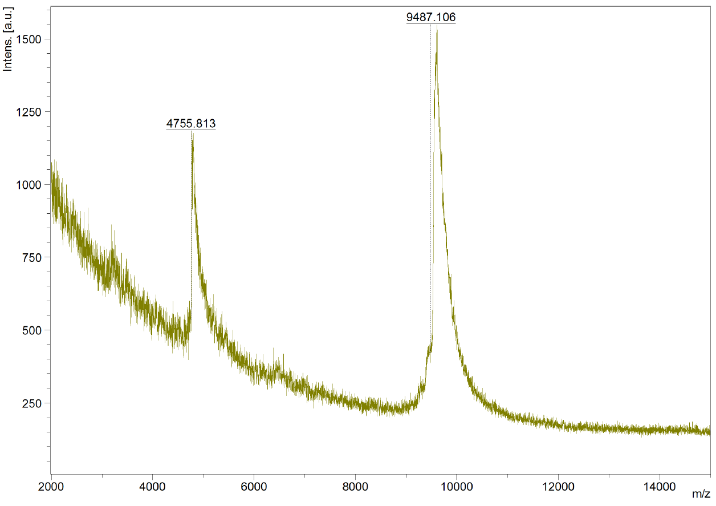
**1) 2)**

**Fig A. Characterization of purified CTD binding linker.** MALDI-TOF spectra (1) and UHPLC spectra (2) of purified CTD binding linker.
