# Supplementary material for: Mutations in microRNA-128-2-3p identified with amplification-free hybridization assay
Source: PLoS One. 2023 Aug 22;18(8):e0289556. doi: 10.1371/journal.pone.0289556 (PMC10443835; doi:10.1371/journal.pone.0289556)
Supplement: S3 File — Sequences of model miRNA targets used in UV thermal denaturation study; Tm results for duplexes of biotinylated capture probes C1-C7 with complementary and mismatched model miRNA targets; Representative UV melting curves for capture probe. (DOCX) [file pone.0289556.s006.docx]

Supporting Information S3 File

**UV melting studies of designed DNA:RNA hybrids**

**Table A.** **Sequences of model miRNA targets used in UV thermal denaturation study.**

| **#** | **Capture sequence,**  **5’->3’** | **Mutation:**  **Position/**  **type** | **RNA model complement code** | **RNA complement sequence, 5’->3’** |
| --- | --- | --- | --- | --- |
| C1 | d(AAAGAG+AC+CG+GT) | None, wt | miR128 M1 | r(ACCGGUCUCUUU) |
| C2 | d(TAAGAG+AC+CG+GT) | 3’ terminal, U>A | miR128 M2 | r(ACCGGUCUCUUA) |
| C3 | d(CAAGAG+AC+CG+GT) | 3’ terminal, U>G | miR128 M3 | r(ACCGGUCUCUUG) |
| C4 | d(GAAGAG+AC+CG+GT) | 3’ terminal, U>C | miR128 M4 | r(ACCGGUCUCUUC) |
| C5 | d(ATAGA+GACC+G+GT) | n-1, U>A | miR128 M5 | r(ACCGGUCUCUAU) |
| C6 | d(ACAGA+GACC+G+GT) | n-1, U>G | miR128 M6 | r(ACCGGUCUCUGU) |
| C7 | d(+AGA+GAGA+CCGGT) | n-1, U>C | miR128 M7 | r(ACCGGUCUCUCU) |

*T*_m_ given in Table B is an average of two independent measurements. Representative curves are shown in Fig A.

**Table B.** ***T*_m_ results for duplexes of biotinylated capture probes C1-C7 with complementary and mismatched model miRNA targets.**

| **Capture probe #/Target #** | **miR-128 M1** | **M2** | **M3** | **M4** | **M5** | **M6** | **M7** | **Δ*T*_m_**** |
| --- | --- | --- | --- | --- | --- | --- | --- | --- |
| **C1 wt** | 62 | 39 | Nct | Nct | 38 | Nct | 57 | 17,3 |
| **C2** | 44 | 61 | Nct | 43 | 39 | 40 | 57 | 16,4 |
| **C3** | 45 | 49 | 61 | 50 | Nct | 40 | 57 | 12,8 |
| **C4** | 55 | 40 | Nct | 55 | 47 | 44 | 56 | 6,6 |
| **C5** | 54 | 41 | Nct | 38 | 61 | 37 | Nct | 18,5 |
| **C6** | 46 | 41 | 45 | 39 | 42 | 55 | 56 | 10,2 |
| **C7** | 40 | Nct | 35 | 38 | 41 | Nct | 64 | 25,5 |
|  |  |  |  |  |  |  |  |  |

Nct = no clear transition. ** Average difference in *T*_m_ for match vs mismatch for duplexes. Only values from clear transition duplexes were used for the calculation.

**Fig A. Representative UV melting curves for capture probes**. a) C1 (wild type); b) C2 (3‘ terminal mismatch); c) C5 (3‘-1 mismatch).
