# Supplementary material for: Mutations in microRNA-128-2-3p identified with amplification-free hybridization assay
Source: PLoS One. 2023 Aug 22;18(8):e0289556. doi: 10.1371/journal.pone.0289556 (PMC10443835; doi:10.1371/journal.pone.0289556)
Supplement: S4 File — Chip design and production; fluorophore and photobleaching study in chip. (DOCX) [file pone.0289556.s007.docx]

Supporting Information S4 File

**Dye study in chip to optimize amplification-free assay**

### Chip design and production

For the fluorophore study, we used microfluidic chips from another related project. The microfluidic chips were made of poly(methyl methacrylate) (PMMA) bonded together with PSA (Pressure-sensitive adhesive) constructed with an inlet hole in first layer, main chamber in second and outlet hole in third layer (Fig A1) and measure 1.7 x 14 x 41 mm^3^ (Fig A2). The microfluidic chips were fabricated using laser ablation in 3-layers of 0.5 mm PMMA bonded with 0.1 mm PSA engraved with CO2-laser (Epilog Mini 18 30-watt, Laser cutter). CorelDraw software was applied for drawings for the laser cutter. Prior to cutting, the thin layer of protection plastic was removed from the PMMA surface and the PMMA piece was placed in the epilog on a 1 cm plastic piece to reach the right height for cutting. The outlet - and inlet-layer were engraved from drawings with the settings: 2 x scans, power 30%, speed 20%, 600 DPI. The middle-layer was added 0.1 mm PSA on both sides and engraved with: 3 x scans, 20% power, 20% speed and 600 DPI. The 3 layers of PMMA were combined, aligned and bonded together (20 kN for 1 min), and broken into smaller chips with individual size 1.7 x 14 x 41 mm^3^ and chamber volume of 50 μl as shown in Fig A2.


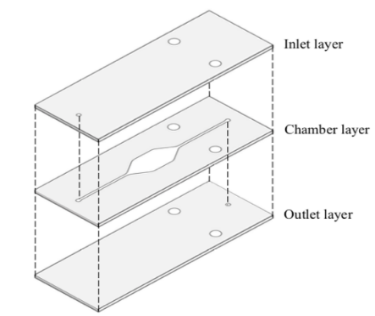


**1) 2)**

14 mm

41 mm

**Fig A. Design of one-chamber microfluidic chip.** 1) Individual layer in the chip aligned. 1) One-chamber chip-metrics; 1.7 x 14 x 41 mm^3^ and chamber volume of 50 μl.

### Fluorophore study in chip

The average fluorescent emission of the duplicates of each dye (EvaGreen (EG), AccuClear (AC), QuantiFluor (QF), Acridine Orange (AO) and Thiazole Orange (TO)) in different concentrations and in the presence of the CTD (booster) are shown in Fig B (1-5). Table A shows the maximum emission of each dye in concentrations: 1.0%, 2.5%, 3.0% and 4.0%.

**Table A.** **Average emission of dyes in comparable dye concentrations.**

|  | **Fluorescent Emission (max)** | | | | |
| --- | --- | --- | --- | --- | --- |
| **Dye conc. %** | **EG** | **AC** | **QF** | **AO** | **TO** |
| **1.0** | 3,11E+04 | 1,53E+05 | 2,01E+05 | 2,96E+05 | 5,90E+05 |
| **2.5** | 5,30E+04 | 3,47E+05 | 4,65E+05 | 3,37E+04 | 3,97E+05 |
| **3.0** | 7,95E+04 | 2,91E+05 | 6,22E+05 | 4,82E+05 | 3,77E+05 |
| **4.0** | 8,05E+04 | 4,96E+05 | 4,77E+05 | 7,14E+05 | 2,40E+06 |
| $\bar{\boldsymbol{X}}$ | 6,10E+04 | 3,22E+05 | 4,41E+05 | 3,82E+05 | 9,41E+05 |

Average emission (max) of the duplicates of EG (525nm), AC (495nm), QF (525nm), AO (525nm) and TO (529nm) in comparable dye concentrations. Mean maximum emission ($\bar{X})$ of the different concentrations was also calculated, showing QF and TO giving the overall highest emission.

**Fig B. Fluorescence emission spectra of five fluorophores in varying concentrations.** 1) EG, excitation (ex): 480 nm 2) AC, ex: 450 nm 3) QF, ex: 480 nm 4) AO, ex: 480 nm and 5) TO, ex: 480 nm. 2 μM CTD was applied.

### Photobleaching study

Photobleaching was calculated in percentage by the formula (1). The mean value ($\bar{X})$ of % photobleaching for each dye in comparable concentrations (1.0%, 2.5%, 3.0%, 4.0%) are shown in Table B. Average max emissions for each dye at 1% concentration are plotted in Fig C.

$100-\left( \frac{max last emission}{max first emission}\cdot100 \right)$ (1)

**Table B.** **The photobleaching of each dye and its corresponding dye concentrations.**

|  | Photobleaching % | | | | |
| --- | --- | --- | --- | --- | --- |
| Dye conc. % | **EG** | **AC** | **QF** | **AO** | **TO** |
| 1.0 | 23.5 | 19.0 | 2.7 | 4,1 | 7,6 |
| 2.5 | 27.4 | 6.9 | 3.6 | -1,5* | 0,3 |
| 3.0 | 25.9 | 5.4 | 1.3 | 22,9 | 6,1 |
| 4.0 | 8.3 | 7.2 | -1.3* | -2,9* | 45,7 |
| $\bar{\boldsymbol{X}}$ | **21.3** | **9.6** | **2.5** | **13.5** | **14.9** |

*Negative values were not part of the mean calculation.

**Fig C. Plotted average max emissions for each dye (1%) over 7 repeated measurements.** Max (EG, AO, QF) = 525nm; max(TO) = 529nm; max(AC) = 495 nm.).

From Table B and Fig C, it is seen from the mean values of % photobleaching over different comparable concentrations and from the slope of the plotted graphs that QF has the overall lowest photobleaching in %. Negative values were not considered in the calculations. Together with the results from the emission study, QF was chosen as the nucleic acid dye for the miRNA detection bead-based assay on its emission qualities and photostability.
