# Supplementary material for: Mutations in microRNA-128-2-3p identified with amplification-free hybridization assay
Source: PLoS One. 2023 Aug 22;18(8):e0289556. doi: 10.1371/journal.pone.0289556 (PMC10443835; doi:10.1371/journal.pone.0289556)
Supplement: S5 File — Calibration curve for RT-qPCR for wild type analyses (miRNA128-2-3p); Concentrations of miRNA128-2-3p and its variants detected in plasma of patients with CRC, colitis and healthy controls using bead-assay and qPCR; Mean CV% for commercial and in house RT-qPCR of miRNA128-2-3p and its mutated variants in position 3’ and 3’-1. (DOCX) [file pone.0289556.s008.docx]

Supporting Information S5 File

**miRNA analysis using amplification-free bead assay and RT-qPCR**

### miRNA analysis

The concentration of miR128 and its variants detected in plasma samples from patients (CRC, colitis and healthy) using bead assay are shown in Tables A-C. The Ct values for obtained from TaqMan RT-qPCR detected miRNA (wt, 3' and 3'-1 variants) were converted to concentrations by use of the standard curve in Fig A and the readouts are shown in Tables E-G. Table D shows the mean CV% for commercial and in house RT-qPCR of miR128-2-3p and its mutated variants in position 3' and 3'-1 for all patient groups.


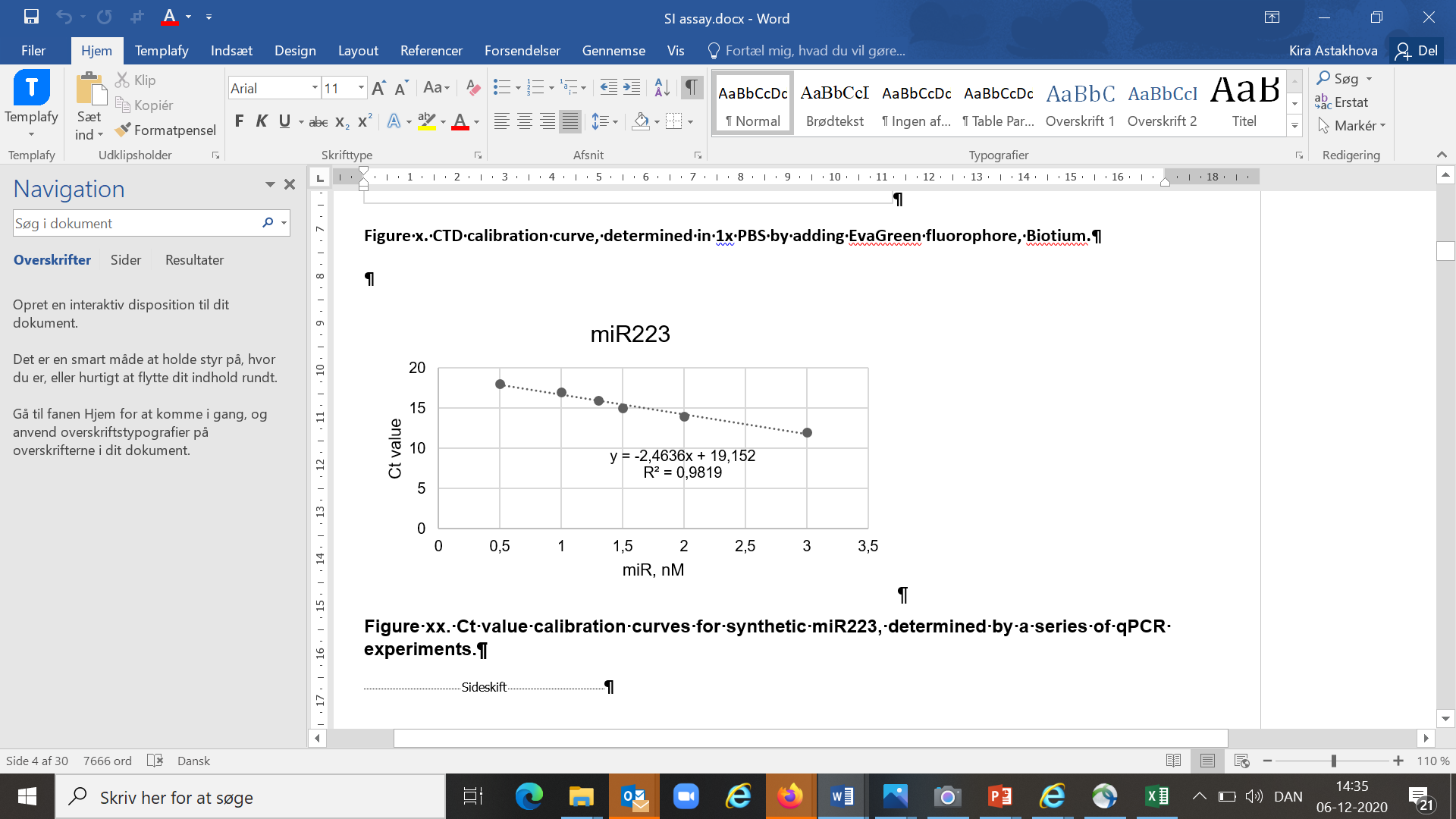


**Fig A. Calibration curve for RT-qPCR for wild type analyses (miR128-2-3p).**

**Table A. Concentrations of miR128 and its variants detected in plasma of patients with CRC using amplification-free bead assay.**

| **pat #/miR128, nM, C#** | **C1** | **C2** | **C3** | **C4** | **C5** | **C6** | **C7** | **C8** | **C9** | **C10** | **C11** | **C12** | **C13** | **C14** |
| --- | --- | --- | --- | --- | --- | --- | --- | --- | --- | --- | --- | --- | --- | --- |
| 15 | 1,24 | 0,33 | 0,34 | 0,46 | 0,83 | 0,41 | 0,55 | 0,30 | 0,56 | 0,44 | 0,33 | 0,31 | 0,42 | 0,01 |
| 23 | 0,88 | 0,42 | 0,67 | 0,68 | 0,53 | 0,47 | 0,29 | 0,45 | 0,81 | 0,36 | 0,47 | 0,50 | 0,38 | 0,01 |
| 41 | 1,45 | 0,58 | 0,60 | 0,40 | 0,36 | 0,48 | 0,72 | 0,41 | 0,56 | 0,73 | 0,28 | 0,20 | 0,36 | 0,01 |
| 24 | 1,48 | 0,44 | 0,30 | 0,36 | 0,66 | 0,57 | 0,66 | 0,47 | 0,83 | 0,36 | 0,27 | 0,24 | 0,22 | 0,01 |
| 54 | 0,55 | 0,50 | 0,82 | 0,63 | 0,70 | 0,71 | 0,69 | 0,33 | 0,32 | 0,46 | 0,23 | 0,50 | 0,32 | 0,01 |
| 66 | 0,28 | 0,30 | 0,30 | 0,32 | 0,73 | 0,33 | 0,36 | 0,52 | 0,66 | 0,36 | 0,31 | 0,41 | 0,24 | 0,00 |
| 73 | 1,07 | 0,55 | 0,46 | 0,41 | 0,81 | 0,82 | 0,69 | 0,36 | 0,39 | 0,71 | 0,44 | 0,20 | 0,46 | 0,01 |
| 111 | 0,59 | 0,31 | 0,50 | 0,36 | 0,48 | 0,70 | 0,67 | 0,42 | 0,56 | 0,62 | 0,35 | 0,28 | 0,25 | 0,01 |
| 122 | 0,55 | 0,57 | 0,70 | 0,65 | 0,50 | 0,32 | 0,80 | 0,43 | 0,58 | 0,32 | 0,28 | 0,36 | 0,30 | 0,01 |
| 132 | 0,74 | 0,53 | 0,67 | 0,60 | 0,65 | 0,75 | 0,61 | 0,80 | 0,37 | 0,79 | 0,52 | 0,19 | 0,22 | 0,01 |
| 140 | 0,33 | 0,37 | 0,32 | 0,82 | 0,67 | 0,73 | 0,54 | 0,38 | 0,80 | 0,79 | 0,44 | 0,45 | 0,20 | 0,02 |
| 118 | 0,28 | 0,41 | 0,57 | 0,69 | 0,71 | 0,37 | 0,47 | 0,43 | 0,79 | 0,57 | 0,52 | 0,32 | 0,26 | 0,01 |
| 156 | 0,81 | 0,34 | 0,70 | 0,43 | 0,59 | 0,66 | 0,64 | 0,76 | 0,64 | 0,31 | 0,53 | 0,41 | 0,15 | 0,01 |
| 189 | 0,82 | 0,54 | 0,63 | 0,36 | 0,63 | 0,65 | 0,50 | 0,35 | 0,53 | 0,61 | 0,40 | 0,19 | 0,17 | 0,00 |
| 221 | 1,63 | 0,60 | 0,47 | 0,72 | 0,35 | 0,53 | 0,72 | 0,80 | 0,58 | 0,80 | 0,35 | 0,18 | 0,35 | 0,02 |
| 212 | 0,96 | 0,32 | 0,70 | 0,47 | 0,57 | 0,39 | 0,47 | 0,77 | 0,87 | 0,47 | 0,55 | 0,27 | 0,20 | 0,01 |
| 234 | 0,66 | 0,52 | 0,41 | 0,39 | 0,38 | 0,76 | 0,86 | 0,32 | 0,59 | 0,82 | 0,30 | 0,36 | 0,21 | 0,01 |
| 356 | 1,06 | 0,62 | 0,71 | 0,63 | 0,29 | 0,74 | 0,88 | 0,52 | 0,78 | 0,55 | 0,38 | 0,16 | 0,47 | 0,01 |
| 401 | 1,39 | 0,35 | 0,67 | 0,29 | 0,78 | 0,32 | 0,41 | 0,81 | 0,60 | 0,35 | 0,48 | 0,36 | 0,18 | 0,01 |
| 408 | 1,11 | 0,58 | 0,37 | 0,66 | 0,52 | 0,49 | 0,40 | 0,52 | 0,58 | 0,63 | 0,17 | 0,23 | 0,27 | 0,00 |

QF standard curve applied to calculate fluorescence signal to concentration. Each data point is an average of two inter-plate replicates; CV average 2.4%.

**Table B. Concentrations of miR128 and its variants detected in plasma of patients with colitis using amplification-free bead assay.**

| **pat #/miR128, nM, C#** | **C1** | **C2** | **C3** | **C4** | **C5** | **C6** | **C7** | **C8** | **C9** | **C10** | **C11** | **C12** | **C13** | **C14** |
| --- | --- | --- | --- | --- | --- | --- | --- | --- | --- | --- | --- | --- | --- | --- |
| 124 | 1,14 | 0,62 | 0,61 | 0,39 | 0,72 | 0,46 | 0,49 | 0,71 | 0,66 | 0,64 | 0,44 | 0,44 | 0,16 | 0,01 |
| 131 | 0,97 | 0,4 | 0,8 | 0,37 | 0,47 | 0,48 | 0,48 | 0,57 | 0,66 | 0,45 | 0,46 | 0,19 | 0,36 | 0,01 |
| 137 | 0,49 | 0,32 | 0,44 | 0,57 | 0,39 | 0,71 | 0,51 | 0,54 | 0,81 | 0,72 | 0,25 | 0,47 | 0,4 | 0,02 |
| 146 | 1,45 | 0,57 | 0,32 | 0,42 | 0,65 | 0,43 | 0,43 | 0,77 | 0,57 | 0,41 | 0,15 | 0,39 | 0,17 | 0 |
| 150 | 0,82 | 0,58 | 0,4 | 0,6 | 0,42 | 0,42 | 0,76 | 0,51 | 0,79 | 0,75 | 0,48 | 0,36 | 0,22 | 0,01 |
| 158 | 0,22 | 0,32 | 0,37 | 0,54 | 0,31 | 0,62 | 0,52 | 0,58 | 0,34 | 0,8 | 0,42 | 0,42 | 0,47 | 0,02 |
| 159 | 0,93 | 0,51 | 0,31 | 0,49 | 0,57 | 0,68 | 0,84 | 0,62 | 0,65 | 0,37 | 0,28 | 0,19 | 0,2 | 0,01 |
| 164 | 0,72 | 0,54 | 0,41 | 0,66 | 0,55 | 0,64 | 0,39 | 0,64 | 0,58 | 0,45 | 0,24 | 0,19 | 0,3 | 0,01 |
| 171 | 0,4 | 0,4 | 0,6 | 0,63 | 0,5 | 0,68 | 0,7 | 0,74 | 0,78 | 0,8 | 0,43 | 0,43 | 0,48 | 0,01 |
| 174 | 0,64 | 0,41 | 0,7 | 0,74 | 0,83 | 0,67 | 0,71 | 0,55 | 0,78 | 0,8 | 0,16 | 0,25 | 0,19 | 0,01 |
| 185 | 0,38 | 0,53 | 0,45 | 0,35 | 0,82 | 0,37 | 0,31 | 0,65 | 0,81 | 0,67 | 0,2 | 0,46 | 0,31 | 0,01 |
| 200 | 0,22 | 0,29 | 0,61 | 0,66 | 0,41 | 0,42 | 0,5 | 0,61 | 0,54 | 0,47 | 0,4 | 0,27 | 0,3 | 0,02 |
| 121 | 0,84 | 0,33 | 0,5 | 0,59 | 0,73 | 0,4 | 0,38 | 0,64 | 0,64 | 0,6 | 0,5 | 0,27 | 0,36 | 0,02 |
| 122 | 1,47 | 0,39 | 0,53 | 0,64 | 0,62 | 0,37 | 0,8 | 0,43 | 0,32 | 0,34 | 0,51 | 0,17 | 0,29 | 0,01 |
| 123 | 0,97 | 0,49 | 0,35 | 0,51 | 0,49 | 0,44 | 0,5 | 0,52 | 0,71 | 0,78 | 0,33 | 0,41 | 0,25 | 0,01 |
| 133 | 1,06 | 0,6 | 0,74 | 0,54 | 0,63 | 0,55 | 0,72 | 0,78 | 0,59 | 0,81 | 0,24 | 0,21 | 0,35 | 0,01 |
| 139 | 0,97 | 0,51 | 0,65 | 0,67 | 0,57 | 0,34 | 0,39 | 0,38 | 0,72 | 0,43 | 0,45 | 0,49 | 0,32 | 0,02 |
| 140 | 0,88 | 0,57 | 0,71 | 0,39 | 0,34 | 0,6 | 0,64 | 0,63 | 0,36 | 0,35 | 0,43 | 0,24 | 0,32 | 0 |
| 141 | 1,31 | 0,39 | 0,31 | 0,4 | 0,79 | 0,74 | 0,77 | 0,41 | 0,71 | 0,61 | 0,47 | 0,14 | 0,37 | 0,02 |
| 145 | 1,64 | 0,39 | 0,31 | 0,49 | 0,39 | 0,45 | 0,31 | 0,47 | 0,32 | 0,32 | 0,26 | 0,39 | 0,35 | 0,02 |
| 147 | 0,31 | 0,42 | 0,35 | 0,71 | 0,27 | 0,73 | 0,69 | 0,49 | 0,47 | 0,79 | 0,22 | 0,46 | 0,4 | 0,01 |
| 186 | 0,15 | 0,6 | 0,72 | 0,79 | 0,48 | 0,61 | 0,77 | 0,7 | 0,54 | 0,58 | 0,42 | 0,39 | 0,44 | 0,01 |
| 191 | 0,34 | 0,46 | 0,66 | 0,77 | 0,45 | 0,77 | 0,34 | 0,88 | 0,71 | 0,42 | 0,44 | 0,39 | 0,16 | 0,01 |
| 201 | 0,51 | 0,55 | 0,6 | 0,79 | 0,71 | 0,31 | 0,52 | 0,72 | 0,73 | 0,42 | 0,47 | 0,21 | 0,49 | 0,01 |

Each data point is an average of two inter-plate replicates; CV average 2.2%.

**Table C. Concentrations of miR128 and its variants detected in plasma of healthy controls using amplification-free bead assay.**

| **pat #/miR128, nM, C#** | **C1** | **C2** | **C3** | **C4** | **C5** | **C6** | **C7** | **C8** | **C9** | **C10** | **C11** | **C12** | **C13** | **C14** |
| --- | --- | --- | --- | --- | --- | --- | --- | --- | --- | --- | --- | --- | --- | --- |
| 1 | 0,36 | 0,40 | 0,27 | 0,41 | 0,36 | 0,50 | 0,45 | 0,18 | 0,32 | 0,38 | 0,36 | 0,25 | 0,30 | 0,00 |
| 2 | 0,23 | 0,41 | 0,29 | 0,16 | 0,21 | 0,31 | 0,47 | 0,30 | 0,44 | 0,36 | 0,21 | 0,32 | 0,17 | 0,00 |
| 3 | 0,23 | 0,34 | 0,25 | 0,22 | 0,27 | 0,22 | 0,37 | 0,43 | 0,33 | 0,39 | 0,24 | 0,18 | 0,37 | 0,00 |
| 4 | 0,54 | 0,43 | 0,48 | 0,42 | 0,46 | 0,37 | 0,42 | 0,23 | 0,26 | 0,23 | 0,31 | 0,46 | 0,33 | 0,00 |
| 5 | 0,14 | 0,58 | 0,42 | 0,45 | 0,40 | 0,28 | 0,20 | 0,31 | 0,20 | 0,16 | 0,32 | 0,50 | 0,50 | 0,00 |
| 6 | 0,07 | 0,56 | 0,42 | 0,48 | 0,19 | 0,20 | 0,28 | 0,21 | 0,26 | 0,32 | 0,32 | 0,36 | 0,27 | 0,00 |
| 7 | 0,36 | 0,37 | 0,48 | 0,17 | 0,43 | 0,25 | 0,45 | 0,18 | 0,24 | 0,19 | 0,42 | 0,32 | 0,38 | 0,00 |
| 8 | 0,16 | 0,38 | 0,26 | 0,19 | 0,46 | 0,47 | 0,29 | 0,36 | 0,46 | 0,49 | 0,50 | 0,45 | 0,47 | 0,00 |
| 9 | 0,14 | 0,40 | 0,44 | 0,37 | 0,43 | 0,43 | 0,43 | 0,35 | 0,44 | 0,33 | 0,17 | 0,38 | 0,32 | 0,00 |
| 10 | 0,22 | 0,49 | 0,51 | 0,32 | 0,36 | 0,22 | 0,48 | 0,51 | 0,46 | 0,40 | 0,22 | 0,24 | 0,29 | 0,00 |
| 11 | 0,10 | 0,65 | 0,31 | 0,22 | 0,30 | 0,14 | 0,50 | 0,49 | 0,24 | 0,23 | 0,45 | 0,33 | 0,22 | 0,00 |
| 12 | 0,08 | 0,33 | 0,35 | 0,45 | 0,19 | 0,38 | 0,19 | 0,52 | 0,38 | 0,18 | 0,23 | 0,43 | 0,15 | 0,00 |
| 13 | 0,27 | 0,59 | 0,33 | 0,44 | 0,40 | 0,37 | 0,46 | 0,30 | 0,15 | 0,29 | 0,21 | 0,44 | 0,38 | 0,00 |
| 14 | 0,23 | 0,45 | 0,34 | 0,51 | 0,39 | 0,16 | 0,22 | 0,48 | 0,27 | 0,44 | 0,35 | 0,28 | 0,38 | 0,00 |
| 15 | 0,56 | 0,64 | 0,29 | 0,25 | 0,18 | 0,17 | 0,28 | 0,39 | 0,17 | 0,16 | 0,47 | 0,24 | 0,17 | 0,00 |
| 16 | 0,28 | 0,31 | 0,48 | 0,33 | 0,32 | 0,24 | 0,17 | 0,33 | 0,51 | 0,51 | 0,14 | 0,27 | 0,34 | 0,00 |
| 17 | 0,22 | 0,60 | 0,30 | 0,27 | 0,33 | 0,34 | 0,27 | 0,23 | 0,32 | 0,27 | 0,23 | 0,29 | 0,23 | 0,00 |
| 18 | 0,34 | 0,53 | 0,37 | 0,41 | 0,30 | 0,42 | 0,49 | 0,16 | 0,35 | 0,25 | 0,32 | 0,15 | 0,27 | 0,00 |
| 19 | 0,42 | 0,61 | 0,22 | 0,49 | 0,44 | 0,31 | 0,34 | 0,16 | 0,36 | 0,31 | 0,27 | 0,28 | 0,14 | 0,00 |
| 20 | 0,37 | 0,39 | 0,26 | 0,36 | 0,40 | 0,37 | 0,17 | 0,41 | 0,51 | 0,50 | 0,52 | 0,15 | 0,33 | 0,00 |

Each data point is an average of two inter-plate replicates; CV average 2.2%.

**Table D. Mean CV% for commercial and in house RT-qPCR of miR128 and its mutated variants in positions 3’ and 3’-1.**

| **Patient group** | **1**  **(wt)** | **2**  **(3’, U>A)** | **3**  **(3’, U>G)** | **4**  **(3’, U>C)** | **5**  **(3’-1, U>A)** | **6**  **(3’-1, U>G)** | **7**  **(3’-1, U>C)** |
| --- | --- | --- | --- | --- | --- | --- | --- |
| **CRC** | 3,4 | 2,3 | 2,7 | 2,9 | 2,8 | 2,7 | 2,9 |
| **Colitis** | 3,3 | 3,6 | 5,3 | 6,2 | 7,0 | 8,1 | 6,5 |
| **Healthy** | 4,5 | 3,1 | 3,9 | 3,4 | 3,2 | 4,4 | 5,6 |

**Table E. Concentrations of miR128 and its variants detected in plasma of patients with CRC using qPCR.**

| **pat #/miR128, nM, mut variant#** | **1** | **2** | **3** | **4** | **5** | **6** | **7** |
| --- | --- | --- | --- | --- | --- | --- | --- |
| 15 | 0,83 | 1,52 | 1,57 | 1,65 | 1,97 | 1,61 | 1,73 |
| 23 | 1,48 | 0,83 | 1,93 | 1,97 | 1,85 | 1,77 | 1,65 |
| 41 | 0,51 | 0,14 | 1,81 | 1,65 | 1,61 | 1,69 | 1,89 |
| 24 | 2,22 | 1,08 | 1,81 | 1,89 | 2,13 | 2,05 | 2,13 |
| 54 | 0,96 | 1,24 | 1,93 | 1,77 | 1,81 | 1,81 | 1,81 |
| 66 | 0,39 | 1,04 | 1,61 | 1,65 | 1,97 | 1,65 | 1,69 |
| 73 | 0,87 | 0,35 | 1,12 | 1,08 | 1,44 | 1,44 | 1,32 |
| 111 | 1,20 | 0,96 | 1,89 | 1,81 | 1,89 | 2,09 | 2,05 |
| 122 | 0,75 | 0,87 | 1,69 | 1,65 | 1,52 | 1,36 | 1,77 |
| 132 | 1,40 | 1,28 | 2,09 | 2,05 | 2,09 | 2,17 | 2,05 |
| 140 | 0,67 | 1,16 | 1,48 | 1,93 | 1,77 | 1,85 | 1,69 |
| 118 | 0,55 | 0,96 | 1,85 | 1,97 | 1,97 | 1,69 | 1,77 |
| 156 | 0,59 | 0,83 | 1,89 | 1,65 | 1,81 | 1,85 | 1,85 |
| 189 | 0,14 | -0,18 | 2,09 | 1,89 | 2,09 | 2,09 | 1,97 |
| 221 | 1,93 | 0,30 | 1,48 | 1,69 | 1,40 | 1,52 | 1,69 |
| 212 | 1,69 | 1,00 | 1,97 | 1,77 | 1,85 | 1,73 | 1,77 |
| 234 | 1,00 | 1,12 | 1,65 | 1,65 | 1,61 | 1,93 | 2,01 |
| 356 | 1,69 | 1,52 | 2,17 | 2,09 | 1,81 | 2,17 | 2,30 |
| 401 | 1,69 | 0,63 | 1,77 | 1,48 | 1,89 | 1,48 | 1,57 |
| 408 | 1,57 | 1,12 | 1,69 | 1,93 | 1,81 | 1,77 | 1,69 |

Each data point is an average of two inter-plate replicates; CV average 2.8%.

**Table F. Concentrations of miR128 and its variants detected in plasma of patients with colitis using qPCR.**

| **pat #/miR128, nM, mut variant#** | **1** | **2** | **3** | **4** | **5** | **6** | **7** |
| --- | --- | --- | --- | --- | --- | --- | --- |
| 124 | 0,71 | 2,17 | 1,40 | 1,40 | 1,32 | 2,26 | 2,50 |
| 131 | 1,57 | 0,51 | 2,05 | 1,69 | 2,91 | 2,54 | 2,34 |
| 137 | 0,59 | 0,35 | 2,66 | 2,54 | 2,91 | 1,89 | 2,54 |
| 146 | 2,17 | 1,16 | 2,22 | 2,42 | 2,09 | 2,50 | 3,39 |
| 150 | 1,20 | 1,24 | 2,50 | 2,54 | 2,50 | 2,66 | 1,85 |
| 158 | 0,35 | 1,24 | 1,69 | 2,50 | 2,66 | 2,58 | 1,81 |
| 159 | 0,71 | 0,43 | 0,96 | 1,12 | 1,20 | 1,32 | 1,44 |
| 164 | 1,52 | 2,13 | 2,01 | 2,54 | 2,13 | 2,38 | 2,50 |
| 171 | 0,63 | 0,83 | 1,61 | 1,65 | 2,91 | 0,06 | 1,20 |
| 174 | 1,32 | 1,40 | 2,13 | 2,38 | 2,87 | 2,38 | 2,62 |
| 185 | 0,71 | 0,71 | 1,61 | 1,52 | 1,93 | 2,05 | 2,26 |
| 200 | 0,51 | 0,96 | 2,38 | 2,99 | 2,30 | 2,50 | 2,34 |
| 121 | 0,63 | 1,00 | 2,30 | 2,17 | 2,62 | 2,30 | 2,05 |
| 122 | 0,91 | 0,10 | 2,01 | 2,09 | 2,09 | 1,89 | 2,22 |
| 123 | 1,32 | 0,06 | 1,40 | 1,93 | 2,13 | 2,09 | 1,52 |
| 133 | 1,77 | 1,04 | 2,01 | 1,85 | 2,34 | 2,50 | 1,97 |
| 139 | 1,28 | 1,36 | 1,57 | 1,36 | 2,34 | 1,61 | 1,65 |
| 140 | 1,85 | 1,32 | 2,17 | 1,89 | 2,22 | 2,54 | 2,09 |
| 141 | 2,26 | 1,89 | 2,05 | 1,57 | 1,89 | 1,85 | 1,89 |
| 145 | 2,09 | 1,32 | 2,26 | 2,54 | 2,46 | 2,54 | 2,38 |
| 147 | 0,67 | 0,26 | 1,40 | 1,69 | 1,32 | 1,73 | 1,69 |
| 186 | 0,96 | 1,28 | 1,97 | 2,05 | 1,81 | 2,54 | 2,42 |
| 191 | 0,67 | 1,44 | 1,04 | 2,54 | 1,69 | 2,83 | 2,78 |
| 201 | 1,20 | 0,91 | 2,50 | 2,22 | 2,38 | 1,85 | 1,12 |

Each data point is an average of two inter-plate replicates; CV average 5.7%.

**Table G. Concentrations of miR128 and its variants detected in plasma of healthy controls using qPCR.**

| **pat #/miR128, nM, mut variant#** | **1** | **2** | **3** | **4** | **5** | **6** | **7** |
| --- | --- | --- | --- | --- | --- | --- | --- |
| 1 | 0,14 | 2,46 | 1,48 | 1,44 | 1,57 | 2,46 | 1,65 |
| 2 | 0,83 | 0,51 | 1,65 | 1,69 | 3,31 | 1,04 | 1,77 |
| 3 | 0,10 | 0,10 | 1,52 | 1,40 | 1,52 | 1,40 | 1,61 |
| 4 | 1,36 | 0,91 | 1,97 | 1,73 | 1,97 | 1,69 | 1,93 |
| 5 | 0,59 | 0,55 | 1,57 | 1,08 | 1,57 | 0,96 | 1,40 |
| 6 | 0,18 | 1,32 | 1,73 | 1,89 | 1,24 | 1,65 | 1,61 |
| 7 | 0,10 | 0,55 | 1,12 | 1,24 | 1,89 | 1,32 | 1,08 |
| 8 | 0,79 | 0,91 | 1,69 | 1,69 | 1,61 | 1,93 | 1,32 |
| 9 | 0,39 | 0,71 | 1,48 | 1,40 | 1,73 | 1,44 | 1,89 |
| 10 | 0,91 | 1,04 | 1,52 | 1,81 | 1,00 | 1,73 | 1,65 |
| 11 | 0,47 | 0,59 | 1,81 | 1,40 | 1,73 | 1,32 | 1,77 |
| 12 | 0,35 | 0,75 | 1,52 | 1,77 | 1,24 | 1,69 | 0,83 |
| 13 | 0,02 | 0,87 | 1,65 | 1,69 | 1,89 | 1,61 | 1,89 |
| 14 | 0,10 | 0,10 | 1,00 | 2,01 | 1,89 | 1,73 | 1,28 |
| 15 | 0,91 | -0,18 | 1,73 | 1,32 | 1,24 | 1,24 | 1,77 |
| 16 | 1,04 | 0,83 | 1,48 | 1,65 | 1,65 | 1,61 | 1,52 |
| 17 | 0,59 | 1,00 | 1,81 | 1,52 | 1,57 | 2,26 | 2,42 |
| 18 | 1,04 | 1,36 | 2,46 | 2,91 | 2,26 | 1,93 | 2,83 |
| 19 | 0,75 | 0,83 | 2,01 | 2,13 | 0,87 | 1,85 | 2,26 |
| 20 | 2,05 | 2,91 | 1,57 | 2,05 | 1,69 | 2,01 | 2,30 |

Each data point is an average of two inter-plate replicates; CV average 4.0%.
