# Supplementary material for: Mutations in microRNA-128-2-3p identified with amplification-free hybridization assay
Source: PLoS One. 2023 Aug 22;18(8):e0289556. doi: 10.1371/journal.pone.0289556 (PMC10443835; doi:10.1371/journal.pone.0289556)
Supplement: S6 File — Linear regression study for amplification-free bead assay vs. qPCR; Pearson coefficients for clinical correlation of amplification-free assay. (DOCX) [file pone.0289556.s009.docx]

Supporting Information S6 File

**Statistical analyses**

**Table A. Linear regression study for amplification-free bead assay vs qPCR.**

| **C1/PCR1** | **C2/PCR2** | **C3/PCR3** | **C4/PCR4** | **C5/PCR5** | **C6/PCR6** | **C7/PCR7** |
| --- | --- | --- | --- | --- | --- | --- |
| 0,66 | -0,02 | 0,26 | 0,47 | 0,19 | 0,29 | 0,10 |

Analysis done for all patient groups – Pearson correlation coefficients.

**Table B. Pearson coefficients for clinical correlation of amplification-free assay.**

|  | **Age (years)** | **Gender** | **Stage** | **History of UC/CD*** | **Hemoglobin (Hgb) g/dL** | **ESR (mm/hour)** | **Race** |
| --- | --- | --- | --- | --- | --- | --- | --- |
| **Age (years)** | 1 |  |  |  |  |  |  |
| **Gender** | 0,04 | 1,00 |  |  |  |  |  |
| **Stage** | 0,11 | -0,14 | 1,00 |  |  |  |  |
| **UCCD history** | -0,06 | -0,03 | -0,05 | 1,00 |  |  |  |
| **Hg** | -0,03 | 0,06 | -0,69 | 0,03 | 1,00 |  |  |
| **ESR** | 0,24 | -0,08 | 0,55 | 0,05 | -0,50 | 1,00 |  |
| **Race** | -0,26 | -0,05 | -0,08 | 0,09 | -0,04 | 0,01 | 1,00 |
| **C1** | 0,09 | 0,13 | **-0,21** | -0,16 | 0,01 | -0,11 | -0,17 |
| **C2** | 0,07 | -0,02 | -0,18 | -0,02 | 0,01 | -0,21 | 0,02 |
| **C3** | 0,13 | -0,12 | 0,00 | 0,05 | -0,02 | -0,13 | -0,09 |
| **C4** | -**0,21** | -0,08 | **-0,23** | 0,09 | **0,29** | -0,15 | 0,06 |
| **C5** | -0,06 | -0,12 | 0,12 | 0,02 | -0,14 | **0,21** | -0,10 |
| **C6** | -0,19 | -0,09 | -0,17 | -0,17 | 0,08 | **-0,34** | **0,26** |
| **C7** | -0,11 | 0,03 | -0,17 | **-0,22** | 0,11 | -0,14 | **0,34** |
| **C8** | 0,15 | -0,01 | -0,13 | **0,38** | 0,16 | 0,00 | 0,10 |
| **C9** | -0,10 | 0,01 | -0,05 | -0,15 | 0,10 | -0,16 | 0,12 |
| **C10** | -0,15 | -0,12 | **-0,23** | -0,02 | **0,27** | **-0,26** | 0,12 |
| **C11** | -0,10 | 0,05 | 0,07 | -0,06 | -0,02 | 0,04 | -0,15 |
| **C12** | **-0,30** | -0,07 | 0,09 | 0,14 | -0,14 | 0,03 | 0,07 |
| **C13** | 0,04 | 0,16 | -0,02 | 0,00 | -0,04 | -0,18 | **0,20** |
