# Supplementary material for: Mutations in microRNA-128-2-3p identified with amplification-free hybridization assay
Source: PLoS One. 2023 Aug 22;18(8):e0289556. doi: 10.1371/journal.pone.0289556 (PMC10443835; doi:10.1371/journal.pone.0289556)
Supplement: S7 File — Probe specificity for mixed synthetic target miRNA; Concentrations of miRNA128-variants (M3, M4, M5, M6) detected in plasma of patients with CRC using qPCR and bead assay; Linear regression for amplification-free bead assay vs. qPCR. (DOCX) [file pone.0289556.s010.docx]

Supporting Information S7 File

## Validation of probe specificity

### **Probe specificity for mixed target miRNA**

To confirm specificity of our designed probes to mutant miRNA targets, we performed the assay using mixtures of the synthetic wild type (M1) and synthetic mutant targets (M2, M5, M8) of selected probes (C1, C2, C5, C8) (Fig A). Total miRNA concentration was kept at 0.5 nM; the assay was performed as previously described. Standard curve (S1 Fig) was applied to convert fluorescent signal to concentration plotted in Fig A. The result confirms high specificity of the probes, with a consistent drop of the signal at increased presence of the mismatched (wild type) miRNA.

**Fig A.** **Probe specificity for mixed synthetic target miRNA.**

1. **Validation of bead-assay using qPCR on selected probes**

To further confirm our results of detected miR128 and its mutation variants in CRC, colitis and healthy samples (Tables A-C in S5 File) using the bead-assay, we performed qPCR on M3, M4, M6 and M7 in CRC samples and compared it to bead-assay. Genscript custom mastermix with mutant-specific PCR primers were applied and conditions were similar to wild-type qPCR (described in S5 File). Calibration curves for each mutant-specific primers (M3, M4, M6, M7) were applied to calculate concentrations shown in Table A. Regression between PCR and bead-assay on M3, M4, M6, M7 mutations of miR128 are shown in Table B.

**Table A. Concentrations of miR128-variants detected in plasma of patients with CRC using qPCR and bead assay [nM].**

| Sample | qPCR result, nM | | | | Bead assay Result, nM; probe C# | | | |
| --- | --- | --- | --- | --- | --- | --- | --- | --- |
| **#** | **3** | **4** | **6** | **7** | **C3** | **C4** | **C6** | **C7** |
| 15 | 1.57 | 1.65 | 1.61 | 1.73 | 0.34 | 0.46 | 0.41 | 0.55 |
| 23 | 1.93 | 1.97 | 1.77 | 1.65 | 0.67 | 0.68 | 0.47 | 0.29 |
| 41 | 1.81 | 1.65 | 1.69 | 1.89 | 0.6 | 0.4 | 0.48 | 0.72 |
| 24 | 1.81 | 1.89 | 2.05 | 2.13 | 0.3 | 0.36 | 0.57 | 0.66 |
| 54 | 1.93 | 1.77 | 1.81 | 1.81 | 0.82 | 0.63 | 0.71 | 0.69 |
| 66 | 1.61 | 1.65 | 1.65 | 1.69 | 0.3 | 0.32 | 0.33 | 0.36 |
| 73 | 1.12 | 1.08 | 1.44 | 1.32 | 0.46 | 0.41 | 0.82 | 0.69 |
| 111 | 1.89 | 1.81 | 2.09 | 2.05 | 0.5 | 0.36 | 0.7 | 0.67 |
| 122 | 1.69 | 1.65 | 1.36 | 1.77 | 0.7 | 0.65 | 0.32 | 0.8 |
| 132 | 2.09 | 2.05 | 2.17 | 2.05 | 0.67 | 0.6 | 0.75 | 0.61 |
| 140 | 1.48 | 1.93 | 1.85 | 1.69 | 0.32 | 0.82 | 0.73 | 0.54 |
| 118 | 1.85 | 1.97 | 1.69 | 1.77 | 0.57 | 0.69 | 0.37 | 0.47 |
| 156 | 1.89 | 1.65 | 1.85 | 1.85 | 0.7 | 0.43 | 0.66 | 0.64 |
| 189 | 2.09 | 1.89 | 2.09 | 1.97 | 0.63 | 0.36 | 0.65 | 0.5 |
| 221 | 1.48 | 1.69 | 1.52 | 1.69 | 0.47 | 0.72 | 0.53 | 0.72 |
| 212 | 1.97 | 1.77 | 1.73 | 1.77 | 0.7 | 0.47 | 0.39 | 0.47 |
| 234 | 1.65 | 1.65 | 1.93 | 2.01 | 0.41 | 0.39 | 0.76 | 0.86 |
| 356 | 2.17 | 2.09 | 2.17 | 2.3 | 0.71 | 0.63 | 0.74 | 0.88 |
| 401 | 1.77 | 1.48 | 1.48 | 1.57 | 0.67 | 0.29 | 0.32 | 0.41 |
| 408 | 1.69 | 1.93 | 1.77 | 1.69 | 0.37 | 0.66 | 0.49 | 0.4 |

**Table B. Linear regression on PCR-bead assay (p-values).**

| **PCR3/C3** | **PCR4/C4** | **PCR6/C6** | **PCR7/C7** |
| --- | --- | --- | --- |
| 0.00392 | 0.0319 | 0.0069 | 0.0358 |

Analysis done for CRC patient group.
