# Supplementary material for: Mutations in microRNA-128-2-3p identified with amplification-free hybridization assay
Source: PLoS One. 2023 Aug 22;18(8):e0289556. doi: 10.1371/journal.pone.0289556 (PMC10443835; doi:10.1371/journal.pone.0289556)
Supplement: Attachment — Submitted filename: response plos.pdf [file pone.0289556.s011.pdf]

Dear Referees and Editor

Thank you very much for your comments which we found useful for improving our manuscript.

Implemented changes are listed below and are highlighted in the revised version of the paper and Supporting information.

Journal requirements:

1. Please ensure that your manuscript meets PLOS ONE's style requirements, including those for file naming. The PLOS ONE style templates can be found at

https://journals.plos.org/plosone/s/file?id=wjVg/PLOSOne_formatting_sample_main_body.pdf and

https://journals.plos.org/plosone/s/file?id=ba62/PLOSOne_formatting_sample_title_authors_affiliations.pdf¨

- PLOS ONE style template has been applied

2. We note that the grant information you provided in the ‘Funding Information’ and ‘Financial Disclosure’ sections do not match.

When you resubmit, please ensure that you provide the correct grant numbers for the awards you received for your study in the ‘Funding Information’ section.

- Corrected.

Reviewer #1:
The manuscript proposed a quantitative detection method for mutated microRNA in human plasma samples. The authors designed an amplification-free assay with rationally designed LNA/DNA probes that can be applied to profile SNP. This manuscript is reasonably organized and the assay has potentials to be used for diagnosing cancer cells. Some issues should be addressed before published:

1. The introduction is a bit lengthy, and it is suggested that can be simplified. For example, the authors can emphasize the advantages of this proposed method compared with conventional assays.

- This has been done in the revised paper.

2. The proposed model included locked nucleic acid (LNA) enriched probes that were designed specifically to target RNA. The author indicated that LNA is a nucleic acid analogue which improves the probes' affinity and specificity. Some experimental data need to be presented to prove it.

- We have now added extra references from our previous LNA work and collaborators results using this model with RNA, DNA and mismatch showing LNA ability to improve target binding and specificity due to Tm increase.

4. The resolution of figures should be improved to show clearly, and the axes inside each figure can be enlarged to show clearly.

- Fig1. Done (Needs to be combined in one page)
- Fig2. Done
- Fig3. Done
- Fig4. Done
- TOC - Done

Figures has been put into PP and exist as tiff.

5. Some assays for SNPs analysis should be introduced, such as Nature Biomedical Engineering, 2022, 6, 957-967; Nature Communications, 2019, 10, 3660; Nature Genetics, 2015, 47, 1212-1219; Angewandte Chemie International Edition, 2014, 53, 2389-2393.

- Nature Biomedical Engineering, 2022, 6, 957-967 - Done
- Nature Communications, 2019, 10, 3660 - Done
- Nature Genetics, 2015, 47, 1212-1219 - Done
- Angewandte Chemie International Edition, 2014, 53, 2389-2393. - Done

- These references are now added and described in the introduction part.

Reviewer #2:
In the manuscript entitled "Mutations in mircroRNA-128-2-3p identified with amplification-free hybridization assay", the authors used a quantitative method to detect mutated miR-128-2-3p SNP in inflammatory colitis and colorectal cancer samples. The study emphasizes the significance of SNP detection in mutant miRNA within patient samples. However, the manuscript exhibits readability issues, including grammar and spelling errors, and lacks appropriate citations. To enhance the manuscript further, the following points should be addressed:

Major Points:
1. The designed C2-C13 DNA/LNA probe may still base pair with any variant of miR-128-2-3p due to the 11-nt base pairing that remains after binding with LNA at 30˚C, leading to a high incidence of false positive reads. To address this issue, the authors can perform in vitro or cell-free assays to validate and quantify the false positive reading bias in their methodology.

- According to our design there is a large difference in affinity for wild type and mismatched probe; this is reported in Table 1. To explore this further we performed analyses of mixed wild-type and mutated RNA targets, now included in Figure S8.1.

2. To establish greater confidence in the application of this method, the authors can employ small RNA sequencing (small RNA-seq) to verify the endogenous expression levels of miRNA-128-2-3p mutants in samples from patients with inflammatory colitis and colorectal cancer.

- This is an excellent point and is an object of our ongoing work that we will publish in the future. In addition to qPCR of wild type miRNA target we now included qPCR data for C3,C4,C6 and C7 in supporting info, Table S8.1 and S8.2.

Minor Points:
1. In line 26, "accroaches" should be corrected to "approaches."
    - This has been corrected
2. In lines 134-135, "Then the beads were washed 3 times using each time 100µl 1X PBS, pH 7.2" can be revised to "The beads were then washed 3 times with 100µl of 1X PBS, pH 7.2."
    - This has been corrected
3. In line 307, "However, is" should be changed to "However, it."
    - This has been corrected
4. In line 281, the first author of the citation "Qui et al" should be corrected to "Qiu."
    - This has been corrected
5. To enhance readability, the authors can provide high-resolution figures.
    - Figures are now in high resolution.
